# Psychological richness as a distinct dimension of well-being: Links to mental, social, and physical health

**DOI:** 10.1371/journal.pone.0326528

**Published:** 2025-06-18

**Authors:** Naoki Konishi, Motohiro Kimura, Ken Kihara, Motoyuki Akamatsu, Minako Hosono, Fumie Sugimoto, Damee Choi, Kohei Fuseda, Toshihisa Sato

**Affiliations:** Human Informatics and Interaction Research Institute, The National Institute of Advanced Industrial Science and Technology (AIST), Tokyo, Japan; The Open University of Israel, ISRAEL

## Abstract

In recent years, well-being research has expanded beyond traditional dimensions, recognizing that a fulfilling life may encompass more than happiness and meaning. We examined the unique contributions of a newly proposed dimension of well-being—psychological richness—to mental, social, and physical health outcomes alongside the established dimensions of hedonic and eudaimonic well-being. We assessed well-being using validated scales that measure life satisfaction, meaning in life, and psychological richness and analyzed data from 11,041 participants. We evaluated health outcomes across mental, social, and physical dimensions using the Subjective Well-being Inventory. Our findings revealed that life satisfaction and meaning in life consistently enhanced health outcomes across most domains. However, psychological richness exhibited a more nuanced profile. Specifically, psychological richness was strongly correlated with positive mental and social health indicators, such as confidence in coping and perceived social support, but also uniquely linked to social isolation and perceived physical symptoms. These results suggest that psychological richness fosters cognitive resilience and social engagement despite potential physical health and social connectedness trade-offs. Notably, individuals high in psychological richness did not report heightened negative emotions, even when experiencing social isolation or physical discomfort, aligning this dimension with other forms of well-being. This study identified psychological richness as an essential addition to well-being models, offering fresh perspectives for tailored well-being interventions.

## Introduction

What constitutes a “good life” or “well-being” has long intrigued scholars. Traditionally, well-being has been conceptualized through two primary frameworks: hedonic and eudaimonic. Hedonic well-being emphasizes pleasure, life satisfaction, and positive affect, focusing on the pursuit of comfort, stability, and the avoidance of pain (e.g., [1,2]). In contrast, eudaimonic well-being centers on meaning, purpose, and self-fulfillment, encouraging individuals to achieve their full potential and align with their values (e.g., [3–6]).

Although valuable, these models may not fully capture fulfilling life experiences, particularly those involving novelty, intellectual engagement, or shifts in perspective. Such experiences are increasingly relevant in today’s interconnected and rapidly changing world. Addressing this limitation, Oishi and colleagues proposed a third dimension of well-being: psychological richness. They argue that a “good life” also includes experiences of novelty, cognitive engagement, and perspective-changing events, which contribute to intellectual growth and the development of wisdom (e.g., [7–10]). Psychological richness emphasizes the importance of mentally stimulating experiences, often involving challenges or even adversity, which deepen one’s understanding of oneself and the world (e.g., [11,12]). By integrating this dimension, researchers may gain a more comprehensive and nuanced understanding of what constitutes a fulfilling life in contemporary society.

### Psychological richness, as a distinct dimension of well-being

Psychological richness offers a distinct pathway to well-being, separating it from the pleasure-oriented hedonic and purpose-driven eudaimonic perspectives. While hedonic well-being emphasizes pleasure and positive affect, and eudaimonic well-being focuses on purpose and self-realization, psychological richness values the accumulation of diverse, intellectually stimulating experiences characterized by novelty, adventure, and cognitive engagement (e.g., [10,13–15]).

Research suggests that psychological richness is associated with personality traits and behaviors that differ significantly from those linked to other well-being dimensions. For instance, while hedonic and eudaimonic well-being are often correlated with more conservative traits—such as political conservatism and a preference for social order and maintaining the status quo—psychological richness aligns with openness to experience, political liberalism, and proactive acceptance of social change [10,15]. Individuals high in psychological richness frequently experience a mix of positive and negative emotions, such as the simultaneous presence of job satisfaction and post-work fatigue in professional settings [14]. Unlike those with high hedonic or eudaimonic well-being, who often seek to avoid loneliness, individuals with high psychological richness embrace solitude, perceiving it as a meaningful opportunity for self-reflection and growth [16].

Given these unique characteristics, psychological richness likely impacts health outcomes in ways distinct from hedonic or eudaimonic well-being, which prior research has consistently shown to positively affect health outcomes [17,18]. This dimension encompasses traits and behaviors that include positive and challenging aspects [11,14], underscoring its duality as a source of unique benefits and stressors. These experiences may foster resilience and cognitive flexibility, potentially influencing mental, social, and physical health outcomes in distinctive ways.

### Health outcomes and well-being dimensions

Health encompasses physical, mental, and social dimensions, which are crucial for understanding the unique impacts of psychological richness (e.g., [19,20]). Extensive research has established positive associations between hedonic and eudaimonic well-being and these dimensions. For instance, hedonic well-being, with its focus on life satisfaction and positive affect, is linked to lower mortality rates, reduced chronic illness, improved mental health resilience, and enhanced perceived social support [17,21,22]. Similarly, eudaimonic well-being, which emphasizes meaning and purpose, has been associated with reduced chronic pain and depressive symptoms, improved health behaviors, lower stress levels, enhanced social engagement, and the maintenance of strong social networks [18,23–25].

Despite the well-documented health benefits of hedonic and eudaimonic well-being, the potential health impacts of psychological richness remain largely unexplored. A psychologically rich lifestyle, characterized by diverse and stimulating experiences, may foster cognitive benefits such as adaptability and intellectual growth. However, its emphasis on complexity and novelty could also introduce stressors, potentially resulting in unique health implications.

If psychological richness demonstrates distinct health associations compared to other well-being dimensions, this will reinforce its role as an essential and unique component of well-being. Exploring these dynamics not only broadens our conceptual understanding of well-being but also uncovers new pathways to health that align with the demands of an increasingly complex and rapidly changing world.

### Research purpose and predictions

The present study investigates the unique contributions of psychological richness to mental, social, and physical health outcomes alongside the traditional dimensions of hedonic and eudaimonic well-being. In alignment with the framework proposed [13] we assessed well-being using three established scales: Satisfaction With Life Scale (hedonic), Meaning in Life Questionnaire (eudaimonic), and psychological richness. Although the Satisfaction With Life Scale and the Meaning in Life Questionnaire do not comprehensively capture the full spectrum of hedonic and eudaimonic well-being, respectively (e.g., [26–28]), they were selected in the present study for consistency with previous research by Oishi and colleagues [14,15], who also used these measures to delineate psychological richness from the two established well-being constructs. Therefore, while acknowledging the conceptual limitations, we prioritized comparability with the literature on psychological richness.

These indicators were then used to predict mental, social, and physical health outcomes, assessed through the Subjective Well-being Inventory (SUBI; [29,30]). This study seeks to advance understanding of well-being by exploring how psychological richness uniquely interacts with mental, social, and physical health. By addressing an underexplored area in well-being research, the study provides insights that may refine existing models to more comprehensively capture the diverse range of fulfilling life experiences. Furthermore, understanding the relationship between psychological richness and health outcomes can inform the development of new intervention strategies to enhance well-being. Specifically, these interventions could emphasize experiences that foster adaptability, cognitive growth, and meaningful social engagement.

Given the exploratory nature of this study, the following predictions are proposed based on the distinct characteristics of psychological richness:

1. **Mental Health**: Individuals with higher psychological richness may experience cognitive resilience and adaptability due to exposure to diverse perspectives [13]. However, their openness to challenges and complex situations (e.g., [11]) may also lead to increased stress and anxiety. Accordingly, while psychological richness is fundamentally associated with positive mental health outcomes, it may also correlate with some negative aspects.
2. **Social Health**: Psychological richness, characterized by robust openness and curiosity [14], may positively influence social health by encouraging the pursuit of novel social interactions. However, individuals with high psychological richness may also prefer solitude [16], potentially leading to increased social isolation. Thus, psychological richness may contribute to positive and negative social health outcomes.
3. **Physical Health**: Hedonic and eudaimonic well-being are strongly associated with physical health benefits, and psychological richness may similarly foster positive effects. However, the unique, stimulating experiences valued by individuals with high psychological richness are also linked to stress, fatigue, and negative emotions [11,14]. Consequently, psychological richness may instead have a detrimental impact on physical health outcomes.

## Method

### Participants

A total of 11,041 Japanese-speaking participants residing in Japan were recruited through an online panel. Participants self-reported demographic information including gender, marital status, and income. Detailed participant characteristics are presented in Table 1.

**Table 1:** Demographic Characteristics of the Sample Population.

Variable	n (%)
**Age**	
< 30	1,063 (9.6)
30 - 44	3,202 (29.0)
45 - 59	4,220 (38.2)
≥ 60	2,556 (23.2)
**Gender**	
Women	5,479 (49.6)
Men	5,509 (49.9)
No response	53 (0.5)
**Marital Status**	4,047 (36.7)
**Household income**	
< 2 million JPY	1,066 (9.6)
2 – < 4 million JPY	2,050 (18.6)
4 – < 6 million JPY	2,046 (18.5)
6 – < 8 million JPY	1,477 (13.4)
8 – < 10 million JPY	998 (9.0)
≥ 10 million JPY	929 (8.4)
No response	2475 (22.4)

### Procedure

Participants were recruited through Bulk Inc. (currently known as MSS Inc.), a commercial online research company in Japan that maintains a large, nationally representative panel of registered monitors. Invitations to participate in the survey were distributed based on age and gender stratification criteria to ensure demographic balance. Participants were informed about the study’s purpose and provided online informed consent before beginning the survey. All participants received compensation in the form of reward points upon completing the survey. The exact monetary value of the points varies depending on the platform through which each participant is registered, and this information is not disclosed to or managed by the research company.

The survey was administered by the research company, and we had access only to anonymized response data. In accordance with institutional policy at the National Institute of Advanced Industrial Science and Technology (AIST), ethics approval was not required because no personally identifiable information was collected. This was confirmed by the AIST ethics committee, which issued a formal waiver. All procedures complied with relevant ethical standards, including the Act on the Protection of Personal Information and the JIS Q 15001 (Personal Information Protection Management Systems). Participants were fully informed of their right to withdraw at any time without consequence, as part of the procedures conducted by the research company. The present study shared data with a previous study [31] by our research group.

### Measures

The following scales were included in the survey conducted by the research company. We had access only to the anonymized responses and did not directly administer the questionnaires. Internal consistency for each scale is reported using Cronbach’s alpha (α).

#### Satisfaction with Life Scale (SWLS).

We assessed Hedonic well-being using the SWLS (32), a 5-item scale (α = .90) rated on a 7-point Likert scale ranging from 1 (*Strongly disagree*) to 7 (*Strongly agree*).

#### Meaning in Life Questionnaire (MLQ).

We assessed eudaimonic well-being using the MLQ [6], which consists of two subscales: MLQ-Presence (5 items, α = .86) and MLQ-Search (5 items, α = .90). Each item is rated on a 7-point Likert scale ranging from 1 (*Strongly disagree*) to 7 (*Strongly agree*). Notably, we excluded the MLQ-Search subscale, which measures a preference for seeking meaning in life, from the present study’s analyses.

#### Psychologically Rich Life Questionnaire (PRLQ).

We assessed psychological richness using the PRLQ [14], a 17-item scale (α = .91) designed to capture experiences of novelty, perspective-shifting, and intellectual engagement. Each item is rated on a 7-point Likert scale ranging from 1 (*Strongly disagree*) to 7 (*Strongly agree*). Sample items include: “My life has been psychologically rich,” “I often feel bored with my life” (reverse-coded), and “My life has been full of unique, unusual experiences.”

#### Subjective Well-Being Inventory (SUBI).

We used the 40-item Subjective Well-Being Inventory (SUBI; [29]) to assess subjective well-being across physical, social, and mental health dimensions. The SUBI includes six subscales assessing mental health: general well-being for positive affect (3 items, α = .78), general well-being for negative affect (3 items, α = .68), inadequate mental mastery (7 items, α = .86), transcendence (3 items, α = .66), confidence in coping (3 items, α = .74), and expectation-achievement congruence (3 items, α = .59). We evaluated social health using four subscales: family group support (3 items, α = .76), social support (3 items, α = .82), primary group concern (3 items, α = .37), and deficiency in social contacts (3 items, α = .56). It is essential to note that the primary group concern subscale was administered only to participants with children or spouses, which resulted in a subset of 7,777 participants from the total sample of 11,041. Although some subscales (e.g., Primary Group Concern: α = .37; Deficiency in Social Contact: α = .56) demonstrated relatively low internal consistency, we retained them given their conceptual relevance and prior validation in Japanese samples [32]. Interpretations involving these subscales should be treated with caution due to limited reliability. We assessed physical health using the perceived ill-health subscale, consisting of 6 items (α = .74). Each item was rated on a 3-point Likert scale: 1 (Disagree), 2 (Neither agree nor disagree), and 3 (Agree). Examples of scale items are provided in Table 2. The validated Japanese version of the SUBI, which was adapted to enhance reliability and cultural relevance, was used in this study [32].

**Table 2:** Subjective Well-Being Inventory Subscales and Sample Items.

	Sample Items
**Mental Health**	
Positive affect (3 items)	Do you feel your life is interesting?
Negative affect (3 items)	Do you feel your life is boring/uninteresting?
Inadequate mental mastery (7 items)	Do you feel disturbed by feeling of anxiety and tension?
Transcendence (3 items)	Do you sometimes experience a joyful feeling of being part of marking as of one large family?
Confidence in coping (3 items)	Do you feel you can manage situations even when they do not turn out as expected?
Expectation-achievement congruence (3 items)	Do you normally accomplish what you want do?
**Social Health**	
Family group support (3 items)	Do you consider your family a source of help to you in finding solutions to most of the problems you have?
Social support (3 items)	Do you feel your friends/relatives would help you out if you were in need?
Primary group concerns (3 item)	Do you sometimes worry about the relationship you and your children have?
Deficiency in social contacts (3 items)	Would you wish to have more friends than you actually have?
**Physical Health**	
Perceived ill-health (6 items)	Do you suffer from pains in various parts of your body?

#### Other measures.

We administered other scales, including the Ten Item Personality Inventory (TIPI-J; [33]), the Response Styles Questionnaire [34], the Hedonic and Eudaimonic Motives for Activities [35], and the Subjective Happiness Scale [36]. We included these scales to explore broader constructs but did not use them in the present analysis.

### Data analysis

Data were analyzed using structural equation modeling (SEM) with the lavaan package [37] in R (Version 3.6.3; [38]). We chose SEM to model the relationships among well-being dimensions—life satisfaction, meaning in life, and psychological richness—as predictor variables for subjective health outcomes, including mental, social, and physical health. Model fit was evaluated using the Comparative Fit Index (CFI), Root Mean Square Error of Approximation (RMSEA), and the Standardized Root Mean Square Residual (SRMR). We considered CFI values ≥ .95, RMSEA values ≤ .06, and SRMR values ≤ .08 as indicative of good model fit [39]. To assess potential multicollinearity among these covariates, we conducted an auxiliary linear regression analysis. The variance inflation factors (VIFs) ranged from 1.16 to 1.50, indicating no substantial multicollinearity. We employed structural equation modeling (SEM) rather than conducting multiple separate regression analyses because SEM allows for simultaneous estimation of associations between multiple predictors and correlated outcome variables. This approach minimizes the risk of inflated Type I error due to multiple testing and accounts for shared variance among outcome domains. All covariates (Age, Gender, Marital Status, Income) were entered at the manifest level. A path diagram illustrating the model structure is provided in the Supporting Information (S3 Fig). Prior to SEM, we computed descriptive statistics and Pearson correlation coefficients for all key variables. These results are presented in the Supporting Information (S1 and S2 Tables).

Demographic variables such as age, gender, marital status, and income were included as covariates to control for their potential influence on the relationships between well-being and health outcomes.

## Results

Descriptive statistics and correlation analyses for demographics, well-being indicators, and health outcomes are presented in the supplementary materials (S1 and S2 Tables). The primary results focus on the structural equation modeling (SEM) analysis, which examines the predictive relationships between the well-being dimensions—life satisfaction, meaning in life, and psychological richness—and health outcomes across mental, social, and physical domains.

We found that the SEM model demonstrated acceptable fit indices (CFI = .990, RMSEA = .075, SRMR = .030), indicating a moderate fit. Table 3 provides the standardized beta coefficients (β), confidence intervals, and significance levels, highlighting each well-being dimension’s unique contributions to various health aspects.

**Table 3:** SEM Results: Associations between Well-Being Dimensions and Health Outcomes adjusted for age, gender, marital status and household income.

	Well-being	β	95% CI	*SE*	*z*	*p*
**Mental health**						
Positive affect	LS	.130	[.120,.140]	.005	24.99	<.001
	ML	.066	[.054,.079]	.006	10.45	<.001
	PR	.161	[.149,.174]	.006	25.06	<.001
Negative affect	LS	−.116	[-.127, -.106]	.005	−21.23	<.001
	ML	−.063	[-.076, -.050]	.007	−9.47	<.001
	PR	−.060	[-.074, -.047]	.007	−8.91	<.001
Insufficient mental mastery	LS	−.061	[-.072, -.049]	.006	−10.54	<.001
	ML	−.049	[-.063, -.035]	.007	−7.02	<.001
	PR	−.014	[-.028,.000]	.007	−1.92	.055
Transcendence	LS	.047	[.037,.058]	.005	8.88	<.001
	ML	.103	[.090,.116]	.007	15.81	<.001
	PR	.126	[.113,.139]	.007	19.13	<.001
Confidence in coping	LS	.029	[.018,.039]	.005	5.45	<.001
	ML	.077	[.064,.089]	.006	12.04	<.001
	PR	.162	[.150,.175]	.006	25.04	<.001
Expectation-achievement congruence	LS	.048	[.038,.058]	.005	9.49	<.001
	ML	.067	[.055,.079]	.006	10.76	<.001
	PR	.107	[.095,.119]	.006	17.01	<.001
**Social health**						
Family group support	LS	.076	[.064,.089]	.006	12.22	<.001
	ML	.047	[.032,.062]	.008	6.15	<.001
	PR	.096	[.081,.112]	.008	12.46	<.001
Social support	LS	.060	[.048,.073]	.006	9.47	<.001
	LS	.052	[.037,.067]	.008	6.68	<.001
	PR	.105	[.089,.120]	.008	13.29	<.001
Primary group concerns	LS	−.063	[-.075, -.050]	.006	−10.11	<.001
	ML	.017	[.002,.032]	.008	2.21	.027
	PR	.034	[.019,.049]	.008	4.39	<.001
Deficiency in social contacts	LS	−.052	[-.063, -.041]	.006	−9.16	<.001
	ML	.019	[.005,.032]	.007	2.73	.006
	PR	.053	[.040,.067]	.007	7.65	<.001
**Physical health**						
Perceived ill-health	LS	−.062	[-.072, -.053]	.005	−12.331	<.001
	ML	−.017	[-.029, -.005]	.006	−2.756	.006
	PR	.019	[.006,.031]	.006	2.959	.003

*Note.* This table presents the standardized beta coefficients (β), 95% confidence intervals (CI), standard errors (*SE*), *z*-scores, and *p*-values from the structural equation modeling (SEM) analysis. *LS* represents life satisfaction, *ML* represents meaning in life, and *PR* represents psychological richness.

**Fig 1:**
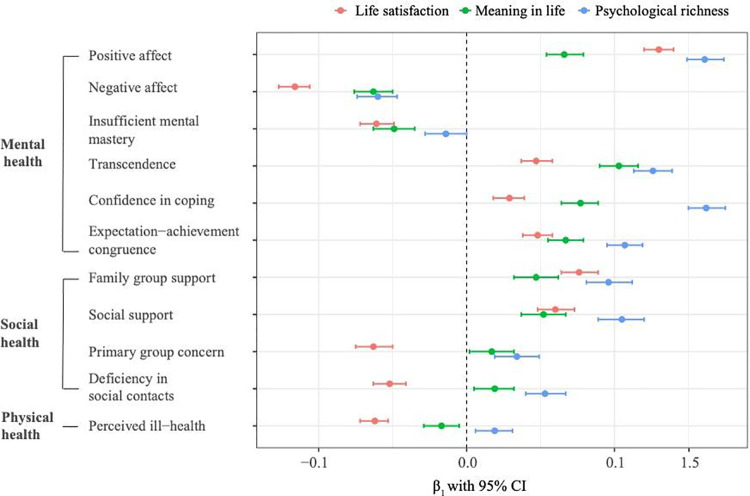
Standardized Beta Coefficients ( β) with 95% Confidence Intervals for the relationships between Well-being Dimensions and Health Outcomes.

Fig 1 illustrates the estimated standardized beta coefficients (β) along with their 95% confidence intervals, showing the relative contributions of each well-being dimension to mental, social, and physical health outcomes. To assess the relative strength of these associations, Fig 1 also examines whether the 95% confidence intervals of standardized beta coefficients overlap. Non-overlapping confidence intervals suggest stronger and more distinct associations between a specific well-being dimension and health outcomes compared to other dimensions.

### Mental health

The results indicate that positive affect was positively associated with all three dimensions of well-being. Psychological richness showed the strongest association (β = 0.161, *p* < .001), followed by life satisfaction (β = 0.130, *p* < .001) and meaning in life (β = 0.066, *p* < .001). In contrast, negative affect was negatively associated with all three dimensions of well-being, with life satisfaction showing the strongest negative association (β = –0.116, *p* < .001), followed by meaning in life (β = –0.063, *p* < .001) and psychological richness (β = –0.060, *p* < .001).

Moreover, insufficient mental mastery, a marker of anxiety and tension, was negatively associated with life satisfaction (β = –0.061, p < .001) and meaning in life (β = –0.049, *p* < .001). However, psychological richness showed a marginally significant relationships (β = –0.014, *p* = .055). Furthermore, transcendence was positively associated with all three well-being dimensions, with psychological richness again showing the strongest association (β = 0.126, *p* < .001), followed by meaning in life (β = 0.103, *p* < .001) and life satisfaction (β = 0.047, *p* < .001). Additionally, confidence in coping exhibited the strongest positive association with psychological richness (β = 0.162, *p* < .001), suggesting its potential role in fostering resilience. Meaning in life (β = 0.077, *p* < .001) and life satisfaction (β = 0.029, *p* < .001) also demonstrated positive associations. Finally, expectation-achievement congruence, reflecting a sense of accomplishment, was positively associated with all three dimensions of well-being. Psychological richness exhibited the most robust relationship (β = 0.107, *p* < .001), followed by meaning in life (β = 0.067, *p* < .001) and life satisfaction (β = 0.048, *p* < .001).

### Social health

For social health indicators, family group and social support were positively associated with all three well-being dimensions. Among these, psychological richness demonstrated significant positive associations with family group support (β = 0.096, *p* < .001) and social support (β = 0.105, *p* < .001). Life satisfaction also showed positive associations with family group support (β = 0.076, *p* < .001) and social support (β = 0.060, *p* < .001), as did meaning in life, though with smaller coefficients (family group support: β = 0.047, *p* < .001; social support: β = 0.052, *p* < .001).

Distinct patterns emerged for primary group concern and deficiency in social contact. Life satisfaction was negatively associated with both primary group concern (β = –0.063, *p* < .001) and deficiency in social contact (β = –0.052, *p* < .001), suggesting that individuals with higher life satisfaction experience fewer relationship concerns and less desire for additional social contact.

In contrast, meaning in life (primary group concern: β = 0.017, *p* = .027; deficiency in social contact: β = 0.006, *p* = .008) and psychological richness (primary group concern: β = 0.034, *p* < .001; deficiency in social contact: β = 0.053, *p* < .001) were positively associated with these indicators. These results suggest that individuals with higher levels of psychological richness or meaning in life may experience greater social isolation or family-related problems.

### Physical health

For physical health, perceived ill-health was negatively associated with both life satisfaction (β = –0.062, *p* < .001) and meaning in life (β = –0.017, *p* = .006), indicating that individuals with higher levels of these well-being dimensions reported fewer physical complaints. In contrast, psychological richness exhibited a unique positive association with perceived ill health (β = 0.019, *p* = .003). This result suggests that individuals high in psychological richness may report more physical symptoms, potentially reflecting the complex and challenging nature of experiences associated with this dimension of well-being.

## Discussion

This study investigated the unique contributions of psychological richness to mental, social, and physical health outcomes compared with life satisfaction (hedonic well-being) and meaning in life (eudaimonic well-being). Individuals with high life satisfaction and meaning consistently scored well on traditional health indicators. However, Psychological richness exhibited a complex and nuanced relationship with health outcomes. These findings underscore psychological richness as a distinct pathway to well-being, characterized by positive impacts and health-related challenges.

### Mental health and well-being dimensions

Consistent with previous research (e.g., [21,23]), the findings indicate that life satisfaction and meaning in life are closely linked to mental health, mainly through their association with reduced negative affect and inadequate mental mastery, indicating anxiety and tension. This finding suggests that managing negative emotions significantly influences traditional forms of well-being, especially hedonic well-being. In contrast, psychological richness shows stronger correlations with indicators like coping confidence, achievement, and positive emotions than other dimensions of well-being.

These stronger associations may stem from the unique life experiences and perspectives associated with higher psychological richness, which enhance individuals’ ability to cope and foster a stronger sense of self-assurance. Specifically, individuals high in psychological richness likely develop confidence through the accumulation of diverse experiences and insights, providing them with the skills and resilience needed to navigate various challenges. Interestingly, in contrast to earlier studies suggesting robust links to heightened negative emotional states (e.g., [11,13], psychological richness displayed only a slight negative correlation with negative affect in the current study. Similarly, a negative relationship with inadequate mental mastery was observed, although it remained a marginal trend.

Although our study, like previous ones, assessed only average levels of negative affect, a possible explanation for this discrepancy is that individuals high in psychological richness may interpret negative experiences as situational rather than enduring. This interpretation remains speculative, but the generally weak association suggests that such individuals do not experience prolonged negative emotions, despite encountering challenging events. Instead, they may perceive such emotions as temporary and context-dependent, contributing to an overall positive emotional state. This highlights the importance of situational framing in understanding the psychological consequences of psychological richness and suggests that interventions could be more effective if they leverage the adaptive coping strategies and resilience commonly associated with this form of well-being.

### Social health and well-being dimensions

Life satisfaction and meaning in life in the current study exhibited positive associations with family and social support, consistent with prior research [22,25]. However, these dimensions of well-being diverged in their relationships with social isolation and family-related concerns. Life satisfaction was negatively associated with social isolation and family-related concerns, suggesting that it fosters close ties and stability in family relationships. In contrast, meaning in life showed weaker positive associations with these factors, suggesting a more ambiguous impact of meaning in life on social isolation and family-related concerns. This suggests that individuals seeking meaning in life may tolerate or even embrace certain difficulties, such as solitude or concerns with family, as part of their broader pursuit of purpose and personal growth.

Interestingly, unlike life satisfaction, psychological richness exhibited a similar yet stronger pattern to meaning in life. Psychological richness was strongly and positively associated with family and social support more than life satisfaction or meaning in life. However, it was also positively related to social isolation and family-related concerns. This finding suggests that individuals high in psychological richness value social connections but seek solitude as an opportunity for self-reflection and perspective-taking. The acceptance of social isolation and family-related concerns in the context of meaning in life is often attributed, at least partially, to the pursuit of purpose and personal growth. In contrast, psychological richness may transcend mere acceptance by framing these challenges as valuable opportunities for gaining new perspectives and enhancing the diversity of one’s experiences [13,16]. For example, social isolation can provide a context for self-reflection and perspective transformation, while family-related challenges may foster deeper relationships and directly contribute to the breadth of experiences integral to psychological richness.

Furthermore, individuals high in psychological richness may actively seek new social connections to fulfill their desire for novel and diverse experiences. Even with supportive relationships, they may feel isolated if familiar friendships lack sufficient stimulation. The ongoing pursuit of novelty might foster social loneliness, even with adequate support. Smith et al. noted that individuals with high psychological richness appreciate solitude, which may reflect their ongoing search for stimulating and meaningful relationships [16]. Thus, they may experience a unique form of social loneliness shaped by the limitations of existing relationships to meet their need for novelty.

Additionally, a degree of family-related challenges may enhance psychological richness by broadening perspectives and deepening life experiences. Individuals high in psychological richness may gravitate toward family and friends who offer social support, as their inclination to seek challenging experiences fosters deeper connections. However, their pursuit of diverse experiences may inadvertently increase family-related worries or concerns. This nuanced interplay between psychological richness, social support, and family-related challenges warrants further investigation to understand its complexities and implications better.

In conclusion, psychological richness overlaps with aspects of meaning in life but is distinguished by its unique emphasis on the “ability and willingness to perceive experiences in diverse and profound ways.” This perspective could be reframed social isolation and family-related concerns as enriching factors that contribute to psychological richness rather than viewing them as solely burdensome. Future research should investigate the mechanisms through which psychological richness is shaped by, and in turn shapes, these experience, thereby providing further insight into its role within the broader framework of well-being.

### Physical health and well-being dimensions

Life satisfaction and meaning in life were associated with having fewer physical symptoms, reflecting their association with conventional health-supportive behaviors (e.g., [17,40]). In contrast, psychological richness showed a slight positive association with perceived ill health, suggesting that individuals high in psychological richness report more physical symptoms. A potential explanation for this finding lies in the inherent trade-offs associated with pursuing psychologically rich experiences. Individuals who seek novelty and cognitive engagement often place themselves in challenging situations that demand significant adaptability and resilience. These experiences, while enriching, can lead to heightened stress and fatigue, which may contribute to physical health costs. Unlike people who prioritize life satisfaction or meaning in life—who are more likely to adhere to conventional health-supportive routines—those with high psychological richness may prioritize novelty and engagement over stability. For example, their irregular sleep patterns or spontaneous travel may inadvertently increase physical health risks.

Exploring the link between psychological richness and specific health behaviors (e.g., sleep, exercise, diet) clarifies how lifestyle factors mediate its relationship with physical health. Given the correlational nature of this study, it is also essential to consider the possibility of a reverse causal relationship. Specifically, physical discomfort may result from diverse life experiences and, in turn, contribute to increased psychological richness by broadening perspectives and understanding of life. Therefore, longitudinal research is needed to establish the directionality of this relationship more definitively.

### Unique relationship between psychological richness and well-being

The findings of this study highlight a critical distinction between life satisfaction and psychological richness in their associations with health outcomes. Individuals with high life satisfaction reported minimal family concerns, robust physical health, and fulfilling social connections, collectively suggesting a stable and harmonious lifestyle that aligns well with conventional health indicators. In contrast, individuals high in psychological richness exhibited a unique profile characterized by a greater likelihood of experiencing family-related concerns, poorer physical health, and social isolation. These findings suggest that psychological richness, unlike life satisfaction, is not necessarily associated with positive health outcomes in the traditional sense. It is widely recognized that individuals who face family worries, long for closer friendships, or experience physical health challenges are often considered “unfortunate.” However, psychological richness may not conform to the conventional notion of the “good life” while fostering a positive outlook and comparable positive emotional states. Notably, despite encountering numerous difficulties and challenges, individuals high in psychological richness exhibited only a slight association with feelings of disappointment, suggesting that they are less likely to perceive life as deeply unsatisfactory. Furthermore, individuals with high psychological richness tended to experience a stronger sense of achievement and confidence in coping with life’s difficulties. This experience may reflect their propensity to pursue challenges [14], providing them with more opportunities to build resilience and develop confidence through successful coping experiences. These insights underscore that psychological richness offers a unique and complex path to well-being. Psychological richness is marked by resilience and personal growth through adversity unlike the stability-focused health benefits associated with life satisfaction and meaning in life.

### Limitations

This study provides valuable insights into the health implications of psychological richness. However, we acknowledge several limitations of the study. First, while the present study adopted the Satisfaction With Life Scale and the Meaning in Life Questionnaire to ensure comparability with prior research on psychological richness, it is important to note that these measures do not encompass the full conceptual scope of hedonic and eudaimonic well-being. The SWLS captures global life satisfaction but does not account for momentary positive and negative affect [1,41], while the MLQ primarily assesses the perceived presence and search for meaning but does not cover broader aspects of human potential and self-actualization as discussed [26]. Therefore, the associations found between psychological richness and the other two well-being constructs should be interpreted with these measurement limitations in mind. Future research would benefit from incorporating broader measures—such as the Positive and Negative Affect Schedule [27] for hedonic well-being and multidimensional assessments of eudaimonia—to more comprehensively map the relationships among well-being constructs [28]. Second, the correlational design of the study restricts the ability to infer causality. Future studies should consider employing longitudinal or experimental designs to understand better the causal pathways underlying the relationships between psychological richness and health outcomes. Third, the cultural context of the sample may limit the generalizability of these findings to other populations, as the participants in this study were exclusively Japanese. Future research should examine potential mediators (e.g., stress regulation mechanisms) and moderators (e.g., cultural and personality differences) to clarify how psychological richness influences health. Such efforts would help determine whether these findings hold across diverse cultural and demographic contexts. Third, the health measures in this study relied exclusively on subjective indicators without incorporating objective health markers. Future research should aim to include objective assessments, such as clinical diagnoses, biological markers of stress or immunity, and physiological indicators, to provide a more comprehensive understanding of the relationship between psychological richness and health outcomes.

Despite these limitations, this study has a notable strength: it employed a large, nationally representative sample of over 11,000 participants, which enhances the generalizability and statistical reliability of the findings. At the same time, the broad age range of participants (18–89 years) may have introduced heterogeneity in health and well-being perceptions. Future studies should explore age-specific trends or conduct stratified analyses to assess potential developmental differences.

## Conclusion

This study highlights psychological richness as a distinct pathway to well-being, diverging from life satisfaction and meaning in life, which align more closely with conventional health indicators. While life satisfaction and meaning in life are associated with reduced negative affect, stronger social ties, and better physical health, psychological richness involves a nuanced trade-off, marked by greater family concerns, physical symptoms, and a tendency toward social isolation. However, it uniquely promotes resilience, coping confidence, and personal growth through diverse and challenging experiences. These findings suggest that psychological richness, while less aligned with traditional dimensions of well-being, offers a transformative and enriching approach to life that emphasizes complexity and growth over stability and comfort.

## Supporting information

S1 Fig Path diagram of the structural equation model. (DOCX)

S1 Table Correlation matrix, means, and standard deviations of well-being indicators. (DOCX)

S2 Table Correlation matrix, means, and standard deviations of health outcomes. (DOCX)

S1 Dataset Fully anonymized dataset used in the analyses. (CSV)
