# Supplementary material for: Psychological richness as a distinct dimension of well-being: Links to mental, social, and physical health
Source: PLoS One. 2025 Jun 18;20(6):e0326528. doi: 10.1371/journal.pone.0326528 (PMC12176124; doi:10.1371/journal.pone.0326528)
Supplement: S1 Fig — (DOCX) [file pone.0326528.s001.docx]

**S1 Fig.** Path diagram of the structural equation model. The model includes psychological richness (PRLQ), life satisfaction (SWLS), and meaning in life (MLQ) as predictors of multiple health outcomes. Covariates (age, gender, marital status, and income) were entered at the manifest level and modeled as predictors of each health outcome. Arrows represent hypothesized paths between variables.
