# Supplementary material for: Psychological richness as a distinct dimension of well-being: Links to mental, social, and physical health
Source: PLoS One. 2025 Jun 18;20(6):e0326528. doi: 10.1371/journal.pone.0326528 (PMC12176124; doi:10.1371/journal.pone.0326528)
Supplement: S1 Table — (DOCX) [file pone.0326528.s002.docx]

**S1 Table**. Correlation Matrix, Means, and Standard Deviations of Well-being Dimensions

|  | Variable | *N* | *M* | *SD* | 1 | 2 |
| --- | --- | --- | --- | --- | --- | --- |
| 1 | Life satisfaction | 11041 | 3.72 | 1.32 |  |  |
| 2 | Meaning in life | 11041 | 3.74 | 1.15 | .59** |  |
| 3 | Psychological richness | 11041 | 4.05 | 1.04 | .48** | .56** |

Note. ** indicates *p* < 0.01
