# Supplementary material for: Psychological richness as a distinct dimension of well-being: Links to mental, social, and physical health
Source: PLoS One. 2025 Jun 18;20(6):e0326528. doi: 10.1371/journal.pone.0326528 (PMC12176124; doi:10.1371/journal.pone.0326528)
Supplement: S2 Table — (DOCX) [file pone.0326528.s003.docx]

**S2 Table**

Correlation Matrix, Means, and Standard Deviations of Mental, Social, and Physical Health

|  |  | *N* | *M* | *SD* | 1 | 2 | 3 | 4 | 5 | 6 | 7 | 8 | 9 | 10 |
| --- | --- | --- | --- | --- | --- | --- | --- | --- | --- | --- | --- | --- | --- | --- |
|  | **Mental health** |  |  |  |  |  |  |  |  |  |  |  |  |  |
| 1 | Positive affect | 11041 | 1.84 | 0.55 |  |  |  |  |  |  |  |  |  |  |
| 2 | Negative affect | 11041 | 1.72 | 0.55 | -.30** |  |  |  |  |  |  |  |  |  |
| 3 | Insufficient mental mastery | 11041 | 1.67 | 0.52 | -.06** | .65** |  |  |  |  |  |  |  |  |
| 4 | Transcendence | 11041 | 1.62 | 0.51 | .63** | -.08** | .13** |  |  |  |  |  |  |  |
| 5 | Confidence in coping | 11041 | 1.78 | 0.51 | .60** | -.18** | -.09** | .56** |  |  |  |  |  |  |
| 6 | Expectation-achievement congruence | 11041 | 1.7 | 0.47 | .56** | -.06** | .09** | .57** | .57** |  |  |  |  |  |
|  | **Social health** |  |  |  |  |  |  |  |  |  |  |  |  |  |
| 7 | Family group support | 11041 | 1.93 | 0.56 | .54** | -.10** | .06** | .50** | .48** | .43** |  |  |  |  |
| 8 | Social support | 11041 | 1.83 | 0.57 | .50** | -.07** | .08** | .51** | .48** | .44** | .63** |  |  |  |
| 9 | Primary group concern | 7777 | 1.72 | 0.51 | .07** | .41** | .45** | .23** | .14** | .20** | .07** | .16** |  |  |
| 10 | Deficiency in social contacts | 11041 | 1.57 | 0.48 | .14** | .42** | .54** | .35** | .16** | .27** | .18** | .23** | .41** |  |
|  | **Physical health** |  |  |  |  |  |  |  |  |  |  |  |  |  |
| 11 | Perceived ill-health | 11041 | 1.59 | 0.43 | .00 | .56** | .65** | .21** | .07** | .17** | .09** | .11** | .42** | .51** |

** indicates *p* < .01
